# Association between in-hospital frailty and health-related quality of life after stroke: the Nor-COAST study

**DOI:** 10.1186/s12883-021-02128-5

**Published:** 2021-03-04

**Authors:** Idunn Snorresdatter Wæhler, Ingvild Saltvedt, Stian Lydersen, Brynjar Fure, Torunn Askim, Marte Stine Einstad, Pernille Thingstad

**Affiliations:** 1 Department of Neuromedicine and Movement Science, Faculty of Medicine and Health Sciences, NTNU-Norwegian University of Science and Technology, Trondheim, Norway; 2 Department of Geriatric Medicine, Clinic of Medicine, St. Olavs Hospital, Trondheim University Hospital, Trondheim, Norway; 3 Department of Mental Health, Faculty of Medicine and Health Sciences, NTNU-Norwegian University of Science and Technology, Trondheim, Norway; 4 Department of Internal Medicine and Department of Neurology, Central Hospital Karlstad and School of Medical Sciences, Örebro University, Örebro, Sweden

**Keywords:** Stroke, Frailty, Older adult, Quality of life, Health-related quality of life

## Abstract

**Background:**

Stroke survivors are known to have poorer health-related quality of life (HRQoL) than the general population, but less is known about characteristics associated with HRQoL decreasing through time following a stroke. This study aims to examine how in-hospital frailty is related to HRQoL from 3 to 18 months post stroke.

**Method:**

Six hundred twenty-five participants hospitalised with stroke were included and followed up at 3 and/or 18 months post stroke. Stroke severity was assessed the day after admission with the National Institutes of Health Stroke Scale (NIHSS). A modified Fried phenotype was used to assess in-hospital frailty; measures of exhaustion, physical activity, and weight loss were based on pre-stroke status, while gait speed and grip strength were measured during hospital stay. HRQoL at 3- and 18-months follow-up were assessed using the five-level version of the EuroQol five-dimensional descriptive system (EQ-5D-5L) and the EuroQol visual analogue scale (EQ-5D VAS). We conducted linear mixed effect regression analyses unadjusted and adjusted for sex, age, and stroke severity to investigate the association between in-hospital frailty and post-stroke HRQoL.

**Results:**

Mean (SD) age was 71.7 years (11.6); mean NIHSS score was 2.8 (4.0), and 263 (42.1%) were female. Frailty prevalence was 10.4%, while 58.6% were pre-frail. The robust group had EQ-5D-5L index and EQ-5D VAS scores at 3 and 18 months comparable to the general population. Also at 3 and 18 months, the pre-frail and frail groups had significantly lower EQ-5D-5L indices than the robust group (*p* <  0.001), and the frail group showed a larger decrease from 3 to 18 months in the EQ-5D-5L index score compared to the robust group (− 0.056; 95% CI − 0.104 to − 0.009; *p* = 0.021). There were no significant differences in change in EQ-5D VAS scores between the groups.

**Conclusion:**

This study on participants mainly diagnosed with mild strokes suggests that robust stroke patients have fairly good and stable post-stroke HRQoL, while post-stroke HRQoL is impaired and continues to deteriorate among patients with in-hospital frailty. This emphasises the importance of a greater focus on frailty in stroke units.

**Trial registration:**

ClinicalTrials.gov (NCT02650531).

## Introduction

Several studies have shown that stroke survivors have impaired health-related quality of life (HRQoL) compared to the general population [1–3]. HRQoL is the subjective quality of an individual’s health status and daily life in terms of physical, mental, and spiritual well-being [4] demonstrated by their expressing satisfaction with their current functional level [5]. HRQoL is affected by patients’ health and function as well as other factors such as cultural background, social life, and environmental features.

Although the incidence of stroke has decreased, the prevalence is expected to rise due to ageing of the population [6]. With improved primary prevention and better treatment in the acute phase—which includes early mobilisation, early medical management, and increased use of recanalization therapies—the mortality rate after stroke has decreased significantly over the past decades [7, 8]. Hence, more people are expected to live with the long-term consequences of stroke [9]. This actualises the need for knowledge about factors related to HRQoL following stroke and how to help people live good lives in the long term.

Previous studies have mainly focused on the impact of post-stroke factors on HRQoL, and they found physical impairment, disability, dependence in ADL, post-stroke depression, cognitive impairment, and age to be the independent factors most commonly influencing HRQoL [1, 2, 10–14].

Lately, there has been increased interest in reduced reserve capacity as a contributing factor to stroke aetiology and functional decline following stroke [15]. Frailty is characterised by reduced physiologic reserve, increased vulnerability to stressors, and multisystem dysregulation [16, 17] with symptoms such as fatigue, decreased strength and endurance, and weight loss [18]. Two previous studies have shown the prevalence of frailty among acute stroke patients to be 24.9% [19] and 28% [20], respectively.

Taylor-Rowan et al. (2019) found pre-stroke frailty to be significantly associated with impaired post-stroke cognition [21], and Landi et al. (2006) found that frail stroke patients presented lower function in activities of daily living (ADL) post-stroke compared to non-frail stroke patients [22]. Moreover, persons with frailty are known to have larger degrees of physical impairment and dependence in ADL and worse HRQoL than the general population [16, 18, 23–26]. Further, low physical functioning and frailty have been associated with a low degree of subjective well-being [27, 28]. This may provide reasons to believe that frail persons will show a lower HRQoL post-stroke than robust individuals.

There is little evidence regarding which patient groups at stroke onset are at risk of experiencing deterioration in HRQoL after stroke. Our hypothesis is that the frail population will have a poorer HRQoL in general and that they will experience a larger deterioration in HRQoL score post-stroke compared to the robust population. More awareness in this field could lead to the development of better and more targeted post-stroke rehabilitation programs focusing on a good life after stroke for exposed patient groups.

The aim of the present study was to investigate whether in-hospital frailty was associated with HRQoL 3 and 18 months after a stroke. Further, we wanted to explore whether frailty was associated with change in HRQoL during the same time period.

## Method

This study is a part of the Norwegian Cognitive Impairment After Stroke study (Nor-COAST), a multicentre prospective cohort study recruiting participants hospitalised with acute stroke in five Norwegian stroke units from May 2015 to March 2017 [29]. Participants had to 1) be admitted to one of the five participating study centres within 7 days after symptom debut, 2) be Scandinavian speaking, 3) be over 18 years old, and 4) live in the catchment area of the recruiting hospitals. Exclusion criterion was expected survival of less than 3 months. Participants were assessed during hospital stay and at 3 and 18 months after the stroke incident at out-patient clinics or by telephone interview. Participants with assessments of HRQoL at either 3 or 18 months were included in the analysis.

### Demographics and clinical information

Demographic information was retrieved from medical records, interviews with participants, and/or by proxy. Information about mortality was collected from participants’ electronic hospital records, which are linked to the National Death Registry. We classified the strokes according to the World Health Organization (WHO) criteria [30] or by findings of acute infarction or cerebral haemorrhage using CT and MRI scans. Stroke severity was assessed at day one post-stroke by the National Institutes for Health Stroke Scale (NIHSS), scoring 0–42 points with a high score indicating a severe stroke [31]. Comorbidity was identified through Charlson Comorbidity Index (CCI) [32]. Information regarding pre-stroke cognition and function was obtained from the patients’ caregivers or close family members during the hospital stay. We used the Global Deterioration Scale (GDS) [33] to assess pre-stroke cognition, while the Montreal Cognitive Assessment (MoCA) [34] was used to assess in-hospital cognitive function. Pre-stroke global function was assessed using the modified Rankin Scale (mRS) [35], and pre-stroke instrumental activities of daily living (i-ADL) was assessed by the Nottingham extended ADL-scale (EADL) [36, 37]. Self-reported data was collected from interview with the participant, or by proxy in case of language impairment or cognitive impairment. Trained health care professionals conducted all interviews and assessments in this study.

### Frailty assessment

To measure frailty at baseline, we used a modified version of the five criteria specified in the Fried phenotype model [18] (Table **1**), including the components exhaustion, unintentional weight loss, low energy expenditure, slow gait speed, and weak grip strength. Information about pre-stroke exhaustion, weight loss, and low physical activity was collected through retrospective self-report from the participant, or by proxy in case of language or cognitive impairment. Gait speed was assessed by measuring the participants’ preferred gait speeds based on the time taken to walk 4 m. Grip strength was evaluated using the value sets of Fried et al. [18], stratified for sex and body mass index (BMI); each participant measured grip strength in each hand three times using a Jamar handheld dynamometer, with the highest value from the strongest hand being used. In the case of a participant not being able to perform an assessment, they were assigned 1point (p) on that criterion, and if there was missing data on a component, the participant was assigned 0 p on that specific criterion, indicating a robust score. A frail state was defined as the presence of three or more criteria (3–5p); a pre-frail state was defined as one or two criteria (1–2p), while absence of criteria (0p) indicated a robust or non-frail state.

**Table 1:** Criteria in our modified version compared to the original Fried phenotype model

Component	Modified version	Original Fried version
**Exhaustion**	Feeling constantly fatigued for more than one week before the stroke	Everything was an effort ≥3 days the last week
**Low physical activity**	Engaging in exercise/ physical activities less than once a week before the stroke	Kilocalories expended per week – lowest quintile^a^
**Weight loss**	Unintentional weight loss of ≥3.0 kg the last 6 months before the stroke	Unintentional loss of ≥4.5 kg OR ≥ 5% of body weight the last year
**Slow gait speed*- Gait test, 4 m***	Duration ≥6 s OR not able	Duration ≥6 s *(women ≥ 159 cm; men > 173 cm)* Duration ≥7 s *(women < 159 cm; men < 173 cm)*
**Weak grip strength –** ***Jamar® dynamometer***	Best measure on strongest hand, using value-sets by Fried et al.^b^ OR not able	Best measure on dominant hand, using value-sets by Fried et al.^b^

*Kcals* Kilocalories; *BMI* Body mass index

^a^ Men < 383 Kcals/week; Women < 270 Kcals/week

^b^ Limits by Fried: Women: BMI ≤ 23.0 or missing BMI, ≤ 17.0 kg; BMI 23.1–26.0, ≤ 17.3 kg; BMI 26.1–29.0, ≤ 18.0 kg; BMI > 29.0, ≤ 21.0 kg;

Men: BMI ≤ 24.0 or missing BMI, ≤ 29.0 kg; BMI 24.1–28.0, ≤ 30.0 kg; BMI > 28, ≤ 32.0 kg

All frailty assessments at the index stay were performed at discharge or on the seventh day of admission for participants with longer hospital stays.

### Quality of life assessment

We used the five-level EuroQol five-dimensional descriptive system (EQ-5D-5L) [38] as a self-reported measure of HRQoL at 3 and 18 months follow-up. The EQ-5D-5L consists of two parts: a five-level descriptive health classifier questionnaire and a visual analogue scale (EQ-VAS).

The EQ-5D-5L questionnaire comprises the five dimensions (5D) mobility, self-care, usual activities, pain/discomfort, and anxiety/depression, each with five levels of response (5 L) from 1p: ‘no problems’ to 5p: ‘extreme problems’*.* Each participant was asked to indicate his/her health state that specific day, choosing the most appropriate statement in each dimension. In the 5 L-questionnaire, the responses for the five dimensions can be combined in a five-digit number describing the participant’s health state, with ‘11111’ meaning no problems in all dimensions to ‘55555’ meaning extreme problems in all dimensions [39]. This health status can be converted into a single summary index. To find the participants’ index scores, we used the EQ-5D-5L Index Value Calculator Version 2.0, developed by the EuroQol Group, utilising the value set from Denmark as there is no value set from Norway to this date. The crosswalk values in this calculator are based on the EQ-5D-3L index calculated by van Hout et al. (2012) [40], with EQ-5D-5L index scores ranging from + 1 to − 0.624, 1 being the best health possible, 0 being dead, and a score <  0 representing a health condition worse than death.

The EQ-VAS provided information about the participants’ subjective health perception: the participants were asked to score their health state that specific day on a visual scale from 0–100p, 0p being ‘the worst health you can imagine’ and 100p being ‘the best health you can imagine’.

Registrations of EQ-5D-5L at 3 and 18 months post-stroke were performed at the outpatient clinic by self-report. Participants unable to attend the outpatient clinics were assessed through telephone interviews.

### Analysis

We present descriptive statistics for the study population in terms of socio-demographic characteristics and pre-stroke clinical characteristics of physical and cognitive function, both in the total population and for the separate frailty groups. Categorical variables are presented as frequencies and percentages, and continuous variables are presented as means and standard deviations (SD). A Kruskal-Wallis test was used for continuous variables, and a linear-by-linear association test was used for categorical variables.

We analysed differences in EQ-5D-5L index and EQ-5D VAS and EQ-5D-5L dimensions between frailty groups at 3 and 18 months, as well as changes over time, using linear mixed effect regression. We used EQ-5D-5L index and EQ-5D-VAS and EQ-5D-5L dimensions as dependent variable, frailty category, and time between 3 and 18 months, respectively; we also used their interaction as categorical covariates and participant as random effect. We did this unadjusted and adjusted for sex, age, and NIHSS score. In the linear mixed effect regression analyses, participants with missing data at one of the time points contributed with data from the available time point. Data at 18 months are regarded as missing for participants who died before 18 months. This way of handling missing data is unbiased when data are missing at random (MAR), while analyses excluding participants with partially missing data (complete case analysis) would be unbiased only under the more restrictive missing-completely-at-random (MCAR) assumption. Normality of residuals was checked by visual inspection of QQ-plots. Statistical significance was defined as a two-sided *p*-value less than 0.05, and we report 95% confidence intervals (CI) where relevant. Analyses were conducted using SPSS 25.

## Results

A total of 815 participants with acute stroke were included in the Nor-COAST study, of whom 625 (76.7%) had measures on the EQ-5D-5L index at 3 and/or 18 months and were included in the analyses. Of these, 578 (92.5%) had measures at 3 months; 493 (78.9%) had measures at 18 months; 446 (71.4%) had measures at both 3 and 18 months, while 132 (21.2%) and 47 (7.5%) had measures only at 3 months and 18 months, respectively.

**Fig. 1:**
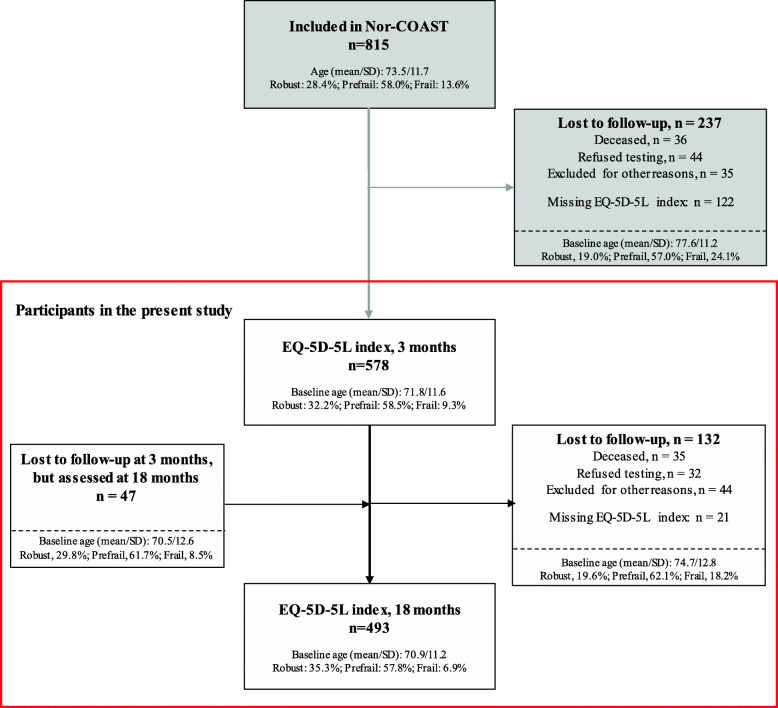
Flowchart of participants included in this study. The black frame represents the participants included in the Nor-COAST study, and the red frame represents the analyses in the present study

Figure 1 presents the flow of subjects analysed in this study. The main reasons for dropout were death, withdrawal from the study, and missing measures on the EQ-5D-5L index. Participants lost to follow-up had a higher prevalence of pre-frail and frail status than those who remained in the study.

### Demographics and clinical data

Table 2 presents demographic and clinical data for the study population. Mean (SD) age was 71.7 (11.6) years; mean NIHSS score was 2.8 (4.1), and pre-stroke mRS-score was 0.8 (1.0), 263 (42.1%) were female. The robust population was younger, comprised of fewer females, had fewer comorbidities (CCI), better pre-stroke physical condition (mRS and EADL scores) and better pre-stroke cognition, suffered from milder strokes, and had better in-hospital MoCA scores compared to the pre-frail and frail population; they more seldom lived alone and had less home nursing prior to the stroke.

**Table 2:** Baseline characteristics

	N	Total	Robust	Pre-frail	Frail	*p*-value^a^
Participants – n (%)	625	625 (100)	194 (31.0)	366 (58.6)	65 (10.4)	
Age						
Mean (SD)	625	71.7 (11.6)	65.6 (11.6)	73.3 (10.6)	81.1 (7.2)	< 0.001
Range		33–96	34–92	33–96	58–95	
Sex						
Female	625	263 (42.1)	53 (27.3)	170 (46.4)	40 (61.5)	< 0.001
Racial category						
Caucasian	624	615 (98.6)	192 (99.5)	360 (98.4)	63 (96.9)	0.117
Education (years)						
Mean (SD)	625	12.4 (3.8)	13.7 (3.5)	12.1 (3.8)	10.2 (3.1)	< 0.001
Living condition pre-stroke						
Own home without home nursing	625	579 (92.6)	193 (99.5)	343 (93.7)	43 (66.2)	< 0.001
Own home with home nursing		44 (7.0)	0 (0)	23 (6.3)	21 (32.3)	
Living alone	625	207 (32.5)	42 (21.6)	125 (34.2)	36 (55.4)	< 0.001
Comorbidities						
Previous cerebral stroke, n (%)	624	110 (17.6)	33 (17.0)	65 (17.8)	12 (18.5)	0.958
Previous TIA, n (%)		29 (4.6)	9 (4.5)	16 (4.4)	4 (6.2)	0.820
Dementia, n (%)		8 (1.3)	2 (1.0)	3 (0.1)	3 (4.6)	0.040
Heart failure, n (%)		20 (3.2)	2 (1.0)	12 (3.3)	6 (9.2)	0.005
COPD, n (%)		31 (5.0)	5 (2.6)	18 (4.9)	8 (12.3)	0.008
Cancer, total, n (%)		98 (15.7)	20 (10.4)	69 (18.8)	9 (13.8)	0.029
Charlson Comorbidity Index						
Mean (SD)	625	3.8 (2.0)	2.9 (1.8)	4.1 (1.9)	5.0 (1.7)	< 0.001
mRS – pre-stroke						
Mean (SD)	621	0.8 (1.0)	0.4 (0.6)	0.8 (0.9)	1.8 (1.3)	< 0.001
≥ 2 points, n (%)		396 (63.8)	81 (42.0)	257 (70.4)	58 (89.2)	
Nottingham EADL – pre-stroke						
Mean (SD)	619	57.6 (10.4)	62.0 (5.5)	57.1 (10.2)	46.9 (13.8)	< 0.001
GDS - pre-stroke						
Mean (SD)	619	1.4 (0.8)	1.1 (0.4)	1.5 (0.8)	2.0 (1.2)	< 0.001
≥ 3 points, n (%)		64 (10.3)	3 (1.6)	41 (11.3)	20 (30.8)	
Stroke classification						
Cerebral infarction	625	574 (91.8)	182 (93.8)	331 (90.4)	61 (93.8)	0.545
Cerebral haemorrhage		51 (8.2)	12 (6.2)	35 (9.6)	4 (6.2)	
NIHSS, day 1						
Mean (SD)	611	2.8 (4.0)	1.8 (3.9)	3.1 (4.0)	4.0 (3.9)	< 0.001
0–4 points, n (%)		500 (81.8)	178 (93.7)	279 (78.2)	43 (67.0)	
5–15 points, n (%)		99 (16.2)	9 (4.7)	69 (19.3)	21 (32.8)	
16–20 points, n (%)		7 (1.1)	1 (0.5)	6 (1.7)	0 (0)	
> 20 points, n (%)		5 (0.8)	2 (1.0)	3 (0.8)	0 (0)	
MoCA – in-hospital						
Mean (SD)	571	23.5 (5.0)	25.4 (3.9)	23.2 (4.9)	19.4 (5.3)	< 0.001

N is the number of participants with data on the required test or question

All measures are given as n (%) unless otherwise stated

^a^Linear-by-linear associations for dichotomous variables; Kruskal-Wallis test for continuous variables;

*COPD* Chronic Obstructive Pulmonary Disease; *NIHSS* National Institute of Health Stroke Scale, range 0-34p; *mRS* Modified Rankin Scale, range 0-6p; *GDS* Global Deterioration Scale; range 0-7p; *MoCA* Montreal Cognitive Assessment, range 0-30p; *Nottingham EADL* Nottingham Extended Activities of Daily Living scale, range 0-66p

The frailty distribution in our study population comprised of 194 robust (31.0%), 366 pre-frail (58.6%), and 65 frail (10.4%) participants. Slow gait speed was the most common symptom with *n* = 217 (36.4%), while weight loss was the least common symptom with *n* = 67 (10.9%). In total, 74 participants were missing data on Fried components. Table 3 presents the distribution of the modified Fried criteria.

**Table 3:** Distribution of modified Fried criteria among the participants

Component	Operational definition	N	Total	Prefrail	Frail
Exhaustion	*Q1*: ‘Did you feel constantly fatigued for more than one week before the stroke?’	613	115 (18.7)	80 (22.2)	35 (54.7)
Low physical activity	*Q2:* ‘Did you engage in exercise/physical activities less than once a week before your stroke?’	617	126 (20.4)	85 (23.4)	41 (63.1)
Weight loss	*Q3:* ‘Have you experienced unintentional weight loss of 3 kg or more in the last 6 months?’	606	67 (11.1)	46 (12.8)	21 (32.8)
Slow gait speed	*A1:* Gait test 4 m: ≥ 6 s OR not able.	596	217 (36.4)	157 (42.9)	60 (90.9)
Weak grip strength	*A2:* Grip strength limits defined by Fried^a^ OR not able.	565	190 (33.6)	134 (40.4)	56 (84.8)

All measures are given as n (%)

*BMI* Body mass index

^a^Limits by Fried: Women: BMI ≤ 23.0 or missing BMI, ≤ 17.0 kg; BMI 23.1–26.0, ≤ 17.3 kg; BMI 26.1–29.0, ≤ 18.0 kg; BMI > 29.0, ≤ 21.0 kg;

Men: BMI ≤ 24.0 or missing BMI, ≤ 29.0 kg; BMI 24.1–28.0, ≤ 30.0 kg; BMI > 28, ≤ 32.0 kg

This table shows the number and proportion of prefrail/frail participants fulfilling the five criteria. *N* is the number of participants who completed the required test/question. Q1–3 are questions about the pre-stroke state, while A1–2 are physical assessments performed in hospital

### Frailty and HRQoL

**Fig. 2:**
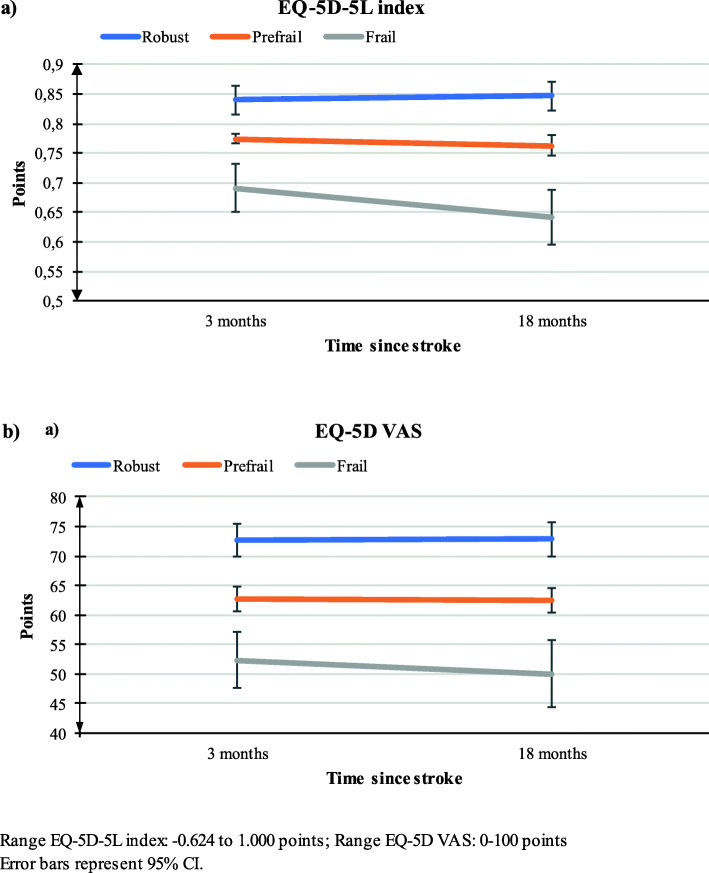
Health-related quality of life scores for the three frailty groups at 3 and 18 months

Results of the unadjusted and adjusted linear mixed effect regression analyses are presented in Tables 4 and 5, respectively. Figure 2 presents change in EQ-5D-5L index and EQ-5D VAS between 3 and 18 months in each frailty group.

**Table 4:** Relationship between frailty group and health-related quality of life score

	ROBUST		PRE-FRAIL				FRAIL			
					Difference from Robust				Difference from Robust	
Unadjusted	N	Mean (95% CI)	N	Mean (95% CI)	Estimate (95% CI)	*p value*	N	Mean (95% CI)	Estimate (95% CI)	*p value*
**3 months**										
EQ-5D-5L index	180	0.865 (0.841 to 0.889)	337	0.767 (0.749 to 0.784)	− 0.098(− 0.128 to − 0.069)	*< 0.001*	61	0.659 (0.618 to 0.701)	−0.206 (− 0.253 to − 0.158)	*< 0.001*
EQ-5D VAS	183	73.6 (71.0 to 76.2)	325	62.4 (60.4 to 64.4)	−11.3 (− 14.5 to −8.0)	*< 0.001*	60	50.8 (46.2 to 55.3)	− 22.9 (− 28.1 to −17.7)	*< 0.001*
**18 months**										
EQ-5D-5L index	168	0.872 (0.847 to 0.896)	284	0.755 (0.737 to 0.773)	−0.117 (− 0.147 to − 0.086)	*< 0.001*	41	0.596 (0.549 to 0.643)	−0.276 (− 0.329 to − 0.223)	*< 0.001*
EQ-5D VAS	168	73.9 (71.2 to 76.6)	272	62.4 (60.3 to 64.4)	−11.5 (− 14.9 to −8.1)	*< 0.001*	38	48.5 (43.0 to 53.9)	−25.4 (− 31.5 to −19.3)	*< 0.001*
**Change between 3 and 18 months**										
EQ-5D-5L index	194	0.007 (−0.014 to 0.028)	366	−0.012 (− 0.005 to 0.028)	−0.019 (− 0.046 to 0.008)	*0.175*	65	− 0.063 (− 0.105 to − 0.020)	−0.070 (− 0.117 to − 0.022)	*0.004*
EQ-5D VAS	194	0.2 (− 2.4 to 2.9)	355	0.0 (− 2.0 to 2.1)	−0.3 (− 3.6 to 3.9)	*0.880*	64	−2.3 (2.7)	−2.5 (− 8.6 to 3.5)	*0.412*
**3 months**										
EQ-5D-5L index	176	0.840 (0.816 to 0.864)	330	0.774 (0.757 to 0.790)	−0.067 (− 0.097 to − 0.037)	*< 0.001*	60	0.691 (0.650 to 0.732)	− 0.149 (− 0.198 to − 0.100)	*< 0.001*
EQ-5D VAS	179	72.6 (69.8 to 75.3)	321	62.7 (60.8 to 64.7)	−9.8 (− 13.3 to − 6.3)	*< 0.001*	59	52.3 (47.6 to 57.1)	−20.2 (− 25.9 to − 14.5)	*< 0.001*
**18 months**										
EQ-5D-5L index	164	0.847 (0.822 to 0.871)	330	0.762 (0.745 to 0.780)	− 0.084 (− 0.115 to 0.054)	*< 0.001*	40	0.641 (0.594 to 0.687)	− 0.206 (− 0.260 to 0.152)	*< 0.001*
EQ-5D VAS	164	72.8 (70.0 to 75.7)	320	62.5 (60.4 to 64.7)	− 10.3 (− 13.9 to − 6.7)	*< 0.001*	37	50.0 (44.3 to 55.7)	−22.8 (− 29.3 to − 16.3)	*< 0.001*
**Change between 3 and 18 months**										
EQ-5D-5L index	194	0.006 (− 0.015 to 0.028)	357	− 0.011 (− 0.028 to 0.005)	− 0.018 (− 0.045 to 0.009)	*0.203*	64	−0.050 (−0.092 to −0.007)	−0.056 (−0.104 to −0.009)	*0.021*
EQ-5D VAS	194	0.2 (− 2.4 to 2.9)	349	−0.2 (− 2.3 to 1.9)	−0.4 (− 3.8 to 3.0)	*0.803*	63	−2.3 (− 7.8 to 3.2)	− 2.5 (− 8.7 to 3.6)	*0.416*

Dependent variable: EQ-5D-5L index and EQ-5D VAS, respectively; categorical covariate: frailty state; random effect: participants

Relationship between frailty group and health-related quality of life score at 3 and 18 months, respectively, and change in score between 3 and 18 months post stroke. Linear mixed effect regression with EQ-5D score as dependent variable, frailty category and time between 3 and 18 months and their interaction as categorical covariates, and participant as random effect

**Table 5:** Relationship between frailty group and EQ-5D-5L dimensions

	ROBUST		PREFRAIL				FRAIL			
					Difference from Robust				Difference from Robust	
	N	Mean (95% CI)	N	Mean (95% CI)	Estimate (95% CI)	*p value*	N	Mean (95% CI)	Estimate (95% CI)	*p value*
**3 months**										
Mobility	189	1.34 (1.22 to 1.47)	334	1.67 (1.58 to 1.76)	0.34 (0.19 to 0.50)	*< 0.001*	62	2.33 (2.11 to 2.55)	0.99 (0.74 to 1.25)	*< 0.001*
Self-care	189	1.12 (1.02 to 1.21)	333	1.25 (1.18 to 1.31)	0.14 (0.03 to 0.25)	*0.014*	62	1.66 (1.51 to 1.82)	0.55 (0.37 to 0.74)	*< 0.001*
Usual Activities	189	1.46 (1.33 to 1.59)	334	1.80 (1.70 to 1.88)	0.36 (0.19 to 0.52)	*< 0.001*	62	2.53 (2.31 to 2.76)	1.09 (0.82 to 1.35)	*< 0.001*
Pain	187	1.61 (1.47 to 1.75)	333	1.91 (1.81 to 2.01)	0.29 (0.12 to 0.46)	*0.001*	62	2.01 (1.77 to 2.25)	0.39 (0.11 to 0.68)	*0.007*
Anxiety	187	1.49 (1.37 to 1.60)	334	1.60 (1.52 to 1.69)	0.12 (−0.03 to 0.26)	*0.118*	61	1.74 (1.54 to 1.94)	0.25 (0.01 to 0.48)	*0.038*
**18 months**										
Mobility	175	1.36 (1.23 to 1.49)	282	1.78 (1.69 to 1.87)	0.43 (0.27 to 0.59)	*< 0.001*	41	2.81 (2.56 to 3.06)	1.45 (1.17 to 1.73)	*< 0.001*
Self-care	175	1.13 (1.04 to 1.23)	283	1.32 (1.25 to 1.39)	0.20 (0.08 to 0.31)	*0.001*	41	1.94 (1.76 to 2.12)	0.81 (0.60 to 1.02)	*< 0.001*
Usual Activities	175	1.42 (1.28 to 1.55)	283	1.73 (1.63 to 1.83)	0.34 (0.17 to 0.51)	*< 0.001*	41	2.64 (2.38 to 2.91)	1.24 (0.93 to 1.54)	*< 0.001*
Pain	173	1.64 (1.50 to 1.79)	281	2.01 (1.91 to 2.12)	0.39 (0.21 to 0.57)	*< 0.001*	41	2.27 (1.99 to 2.55)	0.63 (0.31 to 0.95)	*< 0.001*
Anxiety	175	1.45 (1.33 to 1.56)	282	1.56 (1.48 to 1.65)	0.14 (−0.01 to 0.29)	*0.058*	41	1.74 (1.51 to 1.97)	0.31 (0.04 to 0.57)	*0.022*
**Change**										
Mobility	194	0.02 (−0.09 to 0.14)	366	0.11 (0.02 to 0.20)	0.08 (−0.06 to 0.23)	*0.251*	65	0.48 (0.25 to 0.71)	0.46 (0.20 to 0.71)	*< 0.001*
Self-care	194	0.02 (−0.07 to 0.10)	366	0.07 (0.01 to 0.14)	0.05 (−0.05 to 0.16)	*0.351*	65	0.28 (0.11 to 0.44)	0.26 (0.07 to 0.45)	*0.007*
Usual Activities	194	−0.04 (−0.18 to 0.10)	366	−0.06 (−0.17 to 0.05)	−0.02 (−0.19 to 0.16)	*0.851*	65	0.11 (−0.17 to 0.39)	0.15 (−0.16 to 0.46)	*0.335*
Pain	194	0.02 (−0.13 to 0.16)	366	0.11 (0.00 to 0.23)	0.10 (−0.09 to 0.28)	*0.299*	65	0.26 (−0.03 to 0.54)	0.24 (−0.08 to 0.56)	*0.142*
Anxiety	194	−0.06 (−0.17 to 0.05)	366	−0.03 (−0.12 to 0.06)	0.03 (−0.12 to 0.17)	*0.698*	65	0.00 (−0.23 to 0.23)	0.06 (−0.19 to 0.31)	*0.642*

Dependent variable: Mobility, Self-care, Daily Activities, Pain and Anxiety, respectively. Categorical covariate: frailty state. Random effect: participants

Adjusted for age, sex, and NIHSS-score

Range in each dimension: 1–5 points. Positive value in ‘change’ represents a worsening

Relationship between frailty group and EQ-5D-5L dimensions at 3 and 18 months, respectively, and change in score between 3 and 18 months post stroke. Linear mixed effect regression with EQ-5D-5L dimension as dependent variable, frailty category and time between 3 and 18 months and their interaction as categorical covariates, and participant as random effect

The robust group reported better HRQoL than the pre-frail and frail groups based on EQ-5D-5L index scores and EQ-5D VAS scores at both 3 and 18 months (*p* < 0.001). The robust and the pre-frail groups showed no within-group change in EQ-5D index from 3 to 18 months in either the unadjusted or adjusted model, but there was a decrease in the index score for the frail group (mean change − 0.050, 95% CI − 0.092 to − 0.007, adjusted model) (Table 4).

Compared to the robust group, the frail group had a significant decrease in EQ-5D-5L index score with a between-group difference of − 0.056 (95% CI − 0.104 to − 0.009, *p* = 0.021) in the adjusted model. We noted no between-group difference in change in EQ-5D-5L index between the pre-frail and the robust groups. Furthermore, there were no within-group changes or between-group differences among the three frailty groups as far as change of EQ-5D VAS score in either the unadjusted or the adjusted model (Table 4).

The robust group presented the best scores, and the frail group presented the worst scores in all EQ-5D-5L dimensions at both 3 and 18 months, except for anxiety/depression, which showed no significant difference between the robust and pre-frail groups (Table 5). We also found the frail group to show a larger decrease in mobility and self-care compared to the robust group, with a between-group difference of 0.46 (95% CI 0.20 to 0.71, adjusted) and 0.26 (95% CI 0.07 to 0.45, *p* = 0.007), respectively. We found no significant between-group differences in change between 3 and 18 months between the pre-frail and the robust group in any of the dimensions.

When considering within-group change, the robust group had no significant change in any of the dimensions. The pre-frail and the frail groups showed significant worsening in mobility and self-care (mean [95% CI]: 0.11 [0.02 to 0.20] and 0.07 [0.01 to 0.14]; 0.48 [0.25 to 0.71] and 0.28 [0.11 to 0.44], respectively).

## Discussion

In this descriptive cohort study on Norwegian stroke survivors with mainly minor strokes, we found that frail and pre-frail participants had lower HRQoL than robust participants after 3 and 18 months. HRQoL remained stable for robust and pre-frail participants, while the frail participants showed deterioration in the EQ-5D-5L index from 3 to 18 months post stroke. The EQ-5D VAS score was stable for all groups. Among frail participants, deterioration occurred especially in the dimensions ‘mobility’ and ‘self-care’.

To our knowledge, this is the first study to investigate associations between in-hospital frailty and HRQoL 3 and 18 months after acute stroke. Our hypothesis that frail stroke survivors would have lower HRQoL scores compared to the robust group at both timepoints was confirmed. Based on McClure et al.’s estimations of minimal important difference for six different countries of less than 0.050, there is reason to believe that our finding of a deterioration in EQ-5D-5L index score of 0.050 among the frail participants represents a clinically important effect [41]. This is consistent with findings from non-stroke populations, suggesting a possibly important clinical relationship between frailty and HRQoL [24, 26]. It is also noteworthy that the robust group had HRQoL comparable to a normal population [42–44].

In addition to impaired HRQoL at 3 months, we also found that HRQoL deteriorated from 3 to 18 months among the frail participants in adjusted analyses. The finding of deterioration in the ‘mobility’ and ‘self-care’ dimensions in the pre-frail and frail population is supported by other non-stroke studies showing that frail patients are at risk of experiencing worsening in physical function and ADL [45, 46]. This enhances the importance of identifying patients with frailty and suggests that specific interventions aiming to improve ‘mobility’ and ‘self-care’ should be a topic for future research in order to improve rehabilitation and quality of life for the frail patients.

We also expected the frail population to experience decline in ADL; therefore, it is somewhat surprising that they reported no significant change in the dimension of ‘usual activities’. However, as seen in Table 5, ‘usual activities’ had the least favourable measure at 3 months, showing this dimension to also be associated with poor HRQoL among the frail. Despite adjusting for stroke severity in the analyses, we do not know to what degree the deterioration in HRQoL experienced by the frail participants was a direct consequence of the stroke incident or whether it was a consequence of other mechanisms related to their frailty [47].

Although we found frail participants to experience a decrease in the EQ-5D-5L index, no change was shown in the EQ-5D VAS. There may be several explanations for this. First, the EQ-5D VAS rates the overall health status, including dimensions that are not part of the EQ-5D-5L questionnaire [48]. In addition, older people are more likely to report higher scores in EQ-5D VAS [49], and the frail group had the highest mean age. In addition, EQ-5D VAS has been found to have poor reliability among participants with cognitive impairment [50], and the frail group had a lower MoCA score compared to both the robust and prefrail groups (19.4p vs. 25.4p and 23.2p, respectively), indicating a higher degree of cognitive impairment. Also, post-stroke cognitive impairment was found to be common among the participants in the Nor-COAST study in an additional study by Aam et al. [51]. Finally, frail people tend to better adapt to disability by means of the ‘response shift phenomena’ [52, 53], meaning that while an increased disability will affect a frail person’s EQ-5D-5L index negatively, it may not play any role in the subjective impression of their overall health.

We found 10.4% of the study population to be frail and 58.6% pre-frail in hospital by using a modified version of the Fried phenotype model, while other studies have found higher frailty prevalence and lower pre-frail prevalence in acute stroke populations [19, 20]. A possible explanation for this is that in the present study, participants with missing data on a modified Fried criteria were given 0 points indicating a robust score on that specific criterion, which could partly explain our findings. However, a systematic review by Theou et al. (2015) with 264 studies using the phenotype model to identify frailty, showed a considerable increase of frailty prevalence when imputing missing data with 1 point, as well as an underestimation of frailty prevalence when excluding individuals with at least one missing component from the study [54]. This suggests that missing data could be more indicative of frailty rather than robustness in an individual. Thus, we argue that our method of scoring missing data with 0 points would be a conservative approach. In addition, from baseline to 3 months, 23% of the participants in the Nor-COAST study were excluded from the present study, and 21% were lost to follow-up from 3 to 18 months (Fig. 1). These participants were older with a higher prevalence of frailty and pre-frailty than those who remained in the study. As robust individuals are known to have better HRQoL than frail individuals, the results in the present study therefore likely overestimate EQ-5 L-5D scores and underestimate the decrease in HRQoL from 3 to 18 months.

Of the Fried criteria applied in this study, weight loss, self-reported exhaustion, and low physical activity refer to the participants’ pre-stroke states, while slow gait speed and low grip strength had to be assessed post-stroke and may have been influenced by the stroke incident [55, 56]. We have adjusted for stroke severity, but this may still comprise a methodical challenge in our study*.* Older patients are found more likely to underestimate their disability than younger patients [57, 58]. As studies have shown considerable discrepancies between self-reported function in ADL and actual physical impairment when objectively assessed [59, 60], we would argue that performance-based measures of physical function provide complementary information to self-reports. Considering that stroke is an acute incident, only self- or proxy-reported information about the pre-stroke state of a patient will normally be available for health professionals in a clinical setting, while performance-based measures of physical and cognitive state must be done post stroke. This is a challenge clinicians are facing when identifying frailty in all acute settings, and use of a simple screening tool such as Fried phenotype model would therefore be more feasible compared to more comprehensive assessments as for example a Frailty Index [61] that would embrace broader aspects of frailty. Further discussion on how to best identify patients with frailty in acute settings is of importance and should be a topic for further research.

The major strengths of our study were the large sample size, including more than 600 participants hospitalised with acute stroke from five stroke units in different health regions in Norway, and the high percentage of participants assessed at follow-up with small amounts of missing data.

There are some limitations in this study. First, the study population is slightly younger with smaller strokes and better pre-stroke mRS scores compared to the general Norwegian stroke population [62]; the results are valid for this patient population, meaning that the frailest patients with the most severe strokes have not been included. Secondly, we used the EQ-5D-5L which has been validated for stroke patients [63], but as there is no Norwegian value set available to this date, we used the Danish set being the only Scandinavian version available, and we do not know if this would differ from a Norwegian value set. Lastly, we used a modified version of the Fried criteria that has not been validated. However, both the differences in the groups’ baseline characteristics and clear findings regarding HRQoL are in line with previous research and indicate that our modified version has succeeded in classifying participants as robust, pre-frail, or frail.

## Conclusion

In this study including participants with mainly minor strokes, we found that participants with frailty and pre-frailty reported lower levels of HRQoL at 3 and 18 months post-stroke compared to the robust participants. The robust participants reported fairly good HRQoL that remained stable over time, whereas participants with frailty experienced impaired HRQoL that continued to deteriorate for a long time after the stroke. Especially the functional domains were impaired and continued to deteriorate. Hence, the conception of frailty deserves a larger focus in stroke units in order to provide better personalised treatment, rehabilitation and care planning, and the implementation of routine frailty screening among older patients with acute stroke should be considered.

## Data Availability

The datasets generated and analysed during the current study are not publicly available due to Norwegian regulations and conditions for informed consent, but are available from the corresponding author on reasonable request.

## Abbreviations

ADL: Activities of daily living
BMI: Body mass index
CCI: Charlson comorbidity index
COPD: Chronic obstructive pulmonary disease
CT: Computer tomography
EADL: The nottingham extended activities of daily living scale
EQ-5D-3L: The three-level EuroQol five dimension
EQ-5D-5L: The five-level EuroQol five dimension
EQ-5D VAS: The EeuroQol five-dimension visual analogue scale
GDS: The global deterioration scale
HRQoL: Health-related quality of life
MAR: Missing at random
MCAR: Missing completely at random
MCI: Mild cognitive impairment
MID: Minimal important difference
MMSE: Mini-mental state examination
MoCA: Montreal cognitive assessment
MRI: Magnetic resonance imaging
mRS: Modified rankin scale
NIHSS: National institute of health stroke scale
Nor-COAST: Norwegian cognitive impairment after stroke study
PROMs: Patient reported outcome measures
QoL: Quality of life
WHO: World health Organization
